# The Co-Occurrence of Sexsomnia, Sleep Bruxism and Other Sleep Disorders

**DOI:** 10.3390/jcm7090233

**Published:** 2018-08-23

**Authors:** Helena Martynowicz, Joanna Smardz, Tomasz Wieczorek, Grzegorz Mazur, Rafal Poreba, Robert Skomro, Marek Zietek, Anna Wojakowska, Monika Michalek, Mieszko Wieckiewicz

**Affiliations:** 1 Department of Internal Medicine, Occupational Diseases and Hypertension, Wroclaw Medical University, 50-367 Wroclaw, Poland; helenamar@poczta.onet.pl (H.M.); grzegorzmaz@yahoo.com (G.M.); sogood@poczta.onet.pl (R.P.); ania.wojakowska@wp.pl (A.W.); monika.michalek1@gmail.com (M.M.); 2 Department of Experimental Dentistry, Wroclaw Medical University, 50-367 Wroclaw, Poland; joannasmardz1@gmail.com; 3 Department and Clinic of Psychiatry, Wroclaw Medical University, 50-367 Wroclaw, Poland; dobrewieczorki@gmail.com; 4 Division of Respiratory Critical Care and Sleep Medicine, Department of Medicine, University of Saskatchewan, Saskatoon, SK S7N 5A2, Canada; r.skomro@usask.ca; 5 Department of Periodontology, Wroclaw Medical University, 50-367 Wroclaw, Poland; marekzietek@gazeta.pl

**Keywords:** sexsomnia, sleep sex, sleep bruxism, sleepwalking, sleep talking, obstructive sleep apnea, video-polisomnography

## Abstract

Background: Sleep sex also known as sexsomnia or somnambulistic sexual behavior is proposed to be classified as NREM (non-rapid eye movement) parasomnia (as a clinical subtype of disorders of arousal from NREM sleep—primarily confusional arousals or less commonly sleepwalking), but it has also been described in relation to REM (rapid eye movement) parasomnias. Methods: The authors searched the PubMed database to identify relevant publications and present the co-occurrence of sexsomnia and other sleep disorders as a non-systematic review with case series. Results: In the available literature the comorbidity of sexsomnia and other sleep disorders were reported mainly in case reports and less in case series. Sexsomnia was reported both with one and with multiple sleep-related disorders, with NREM parasomnias and obstructive sleep apnea (OSA) being the most commonly reported. Furthermore, the authors enrich the article with new findings concerning two novel cases of sleep bruxism triggering recurrent sexsomnia episodes. Conclusions: Sexsomnia has still not been reported in the literature as often as other parasomnias. The coexistence of sexsomnia and other sleep-related disorders should be more thoroughly examined. This could help both in sexsomnia as well as other sleep-related disorders management.

## 1. Introduction

Parasomnias comprise a category of sleep disorders with abnormal physical events (movements or behaviors), experiences (dreams, emotions, perceptions), and autonomic nervous system activity. They can appear during entry into sleep, within sleep or during arousals [1,2]. Most parasomnias are dissociated sleep states which are partial arousals during the transitions between wakefulness and non-rapid eye movement (NREM) sleep, or wakefulness and rapid eye movements (REM) sleep [1]. That is why parasomnias can be divided into NREM sleep, rapid eye movement (REM) sleep, and other parasomnias (which usually appear in different sleep stages or during sleep onset or offset) [1]. There are two classifications of sleep disorders: Diagnostic and Statistical Manual of Mental Disorders, fifth edition (DSM-5) classification [3] and the International Classification of Sleep Disorders, 3rd edition (ICSD-3) [2].

Sleep sex is also known as sexsomnia, or somnambulistic sexual behavior is proposed to be classified as a NREM parasomnia (as a clinical subtype of disorders of arousal from NREM sleep—primarily confusional arousals or less commonly sleepwalking), but it has also been described in relation to REM parasomnias [4,5,6,7,8,9,10]. 

Sexsomnia has still not been reported in the literature as often as other parasomnias. The population prevalence is unknown. It can be based only on the estimated overall prevalence of parasomnias, which for adults is estimated to range between 2% and 6% [6]. The aim of this study was to investigate the co-occurrence of sexsomnia and other selected sleep disorders and present it as a non-systematic review with case series, including two novel cases from our center of sleep bruxism triggering recurrent sexsomnia episodes, as documented by video-polysomnography.

## 2. Methods

The authors used the following inclusion criteria: studies that discussed the co-occurrence of sexsomnia and other sleep disorders and behaviours. English language and full-text articles published between January 2003 and June 2018 were included in this non-systematic review. Participants were males and females of any age with a clinical diagnosis of sexsomnia. The authors searched the PubMed database to identify relevant publications. The literature search strategy was conducted as follows: each of two synonymous phrases, i.e., (1) sexsomnia, (2) sleep sex, were combined with each of: (a) sleep disorder, (b) sleep disorders, e.g., “sexsomnia sleep disorder”, viz. (1) + (a); “sexsomnia sleep disorders”, viz. (1) + (b), etc. Four queries were obtained. The authors screened the reference list of included studies to identify other potentially useful studies. Firstly, the authors screened the titles and abstracts, then, the full texts for keywords, such as “sexsomnia”, “sleep sex” to find those that were potentially suitable. The data extraction process focused on the information about each study type and coexisting sleep disorder. The authors also described two clinical cases of patients in whom sexsomnia co-occurred with bruxism and other sleep-related disorders.

## 3. Results

In 1875, Moet reported abnormal sexual activity during sleep for the first time in history [5]. In the available literature, the comorbidity of sexsomnia and other sleep disorders is reported mainly in case reports and less in case series. In some studies, the authors focused more on treatment than the coexistence of sexsomnia with other sleep disorders. Ariño et al. described four cases of sexsomnia. In one patient clonazepam treatment was started and reported to decrease the frequency of both confusional arousals and sexsomnia episodes [11]. Also, Béjot et al. described two cases of sexsomnia in adult women that were successfully treated with selective serotonin reuptake inhibitors (SSRI) [12]. However, a case of SSRI-induced sexsomnia has been also reported [13]. 

### 3.1. Sexsomnia and Obstructive Sleep Apnea/Hypopnea

Obstructive sleep apnea (OSA) is the most common type of sleep apnea and is caused by complete or partial obstructions of the upper airway. It is characterized by repetitive episodes of shallow or paused breathing during sleep, despite the effort to breathe, and is usually associated with a reduction in blood oxygen saturation [2]. The co-occurrence of sexsomnia and obstructive sleep apnea (OSA) was the second most commonly reported, after NREM parasomnias. Meira et al. presented a case of a patient with mild OSA and sexsomnia who had the resolution of both conditions with a mandibular advancement device (MAD) [14]. Another case of successful MAD treatment was described by Khawaja et al. A patient with OSA and sexsomnia with no signs of disordered arousals was treated with MAD, but after five months requested continuous positive airway pressure (CPAP) treatment due to mandibular pain. CPAP treatment turned out to be successful as well [15]. Another case of successful treatment of moderate OSAS and sexsomnia (4-year history before treatment—started together with snoring) was with nasal CPAP as described by Schenck [16]. Successful treatment with nasal CPAP of both disorders was also reported by Della Marca [17]. Two additional cases of such coexistence were described by Ariño, though both patients refused treatment for OSA and sexsomnia [11].

### 3.2. Sexsomnia and Sleep-Related Eating Disorder

Sleep-related eating disorder (SRED) is a condition characterized by recurrent episodes of eating at the transition from night-time sleep to arousal. SRED patients describe eating in an out-of-control manner with a preference for high-caloric foods and sometimes with inedible or toxic items [2,18]. Gomis et al. reported a case of a 41-year-old woman with a diagnosis of narcolepsy with mild cataplexy. After introducing treatment with sodium oxybate, the patient reported sleep-related sexual episodes after which the sleep-related eating episode took place [10].

### 3.3. Sexsomnia and Sleepwalking 

Sleepwalking is also known as noctambulism or somnambulism. It is a phenomenon of combined sleep and wakefulness. It is classified as parasomnia sleep disorder. Yeh et al. reported a case of a 20-year-old soldier presenting sexsomnia and sleepwalking. Researchers tried clonazepam treatment. The treatment controlled the sleepwalking, but not the sleep masturbation episodes [19].

### 3.4. Sexsomnia and Multiple Various Sleep-Related Disorders

In some cases, sexsomnia occurs with not one, but several other sleep-related disorders. Soca et al. presented a case of a 42-year-old male patient with sexsomnia as a part of parasomnia overlap disorder (POD). The patient had sleepwalking, sleep-related eating, confusional arousals, sleep talking, and REM sleep behavior disorder (RBD). In this patient OSA was also diagnosed, which played a promoting role in the sleepwalking and sleep-related eating, as there was a good response to nasal continuous positive airway pressure (nCPAP). Although this therapy did not give an effect on the sexsomnia treatment, it did respond to the bedtime clonazepam therapy [20]. Also, Pelin et al. reported a case of a 31-year-old male who presented sexsomnia with sleepwalking and sleep talking. Sexsomnia, in this case, was treated with carbamazepine [21]. One case presented with a complex history of POD, including sleepwalking, sexsomnia and REM sleep behavior disorder, coexisting with mild OSA. Parasomnias did not respond to nasal CPAP treatment, but there was an improvement after clonazepam administration [22]. Shapiro et al. reported a case of the coexistence of sexsomnia, OSA and sleepwalking, with good response for CPAP. What is more intriguing, the patient ceased therapy because of discomfort—at this moment sexsomnia relapsed, until the treatment reinstitution [23]. Irfan et al. described a case of sleep-related orgasms in a 57-year-old woman who was also diagnosed with hypnic jerks, exploding head syndrome and OSA. In this case, bedtime Clonazepam therapy was effective in sleep-related orgasms and hypnic jerks treatment [24]. Clonazepam therapy was not effective in another case presented by Mioč et al. they described a 33-year-old man presenting episodes of sexsomnia, hypnagogic hallucinations, sleep paralysis, night terrors and sleepwalking. In this patient, further studies led to the diagnosis of sleep-related hypermotor epilepsy. Starting antiepileptic treatment had to be discontinued because of increased violence of the episodes [25].

Data extraction of selected studies is presented in Table 1.

### 3.5. Co-Occurrence of Sexsomnia, Sleep Bruxism and Other Sleep-Related Disorders

The authors will now present two novel case reports that are part of the research project titled “Evaluation of the Quality of Sleep, Endothelial Function, Cardiovascular Risk, Thyroid Function, a Function of Masticatory Muscles and Psycho-emotional State of Patients with Sleep Bruxism”. The project was performed in the Department and Clinic of Internal and Occupational Diseases and Hypertension, Wroclaw Medical University, Wroclaw, Poland. The project was co-financed from funds for young researchers obtained from the Wroclaw Medical University (STM.B022.17.011). The project was accepted by the local Ethical Committee (ID KB-195/2017) and registered on the ClinicalTrials.gov (ID WMU1/2017).

Both of the presented cases show the co-occurrence of sexsomnia and sleep-related bruxism. According to consensus established in 2017 bruxism is a common phenomenon and can be defined as a repetitive jaw-muscle activity characterized by clenching or grinding of the teeth and/or by bracing or thrusting of the mandible. This activity can occur both during sleep time (sleep bruxism (SB)) and during wakefulness (awake bruxism (AB)). Its prevalence ranges from 8 to 31% in the adult population with no gender differences. There are many factors involved in bruxism’s etiology. They can be divided into biological (i.e., neurotransmitters, sleep arousals), psychological (i.e., stress, anxiety, personality) and exogenous factors (i.e., nicotine, alcohol, drugs, medications) [26,27,28,29]. According to the newest commentary to the international consensus on the assessment of bruxism sleep and awake bruxism are generally considered as different behaviors observed during sleep and wakefulness, and that is why the earlier common definition should be separated into two. The new recommended definition of sleep-related bruxism is: “Masticatory muscle activity during sleep that is characterized as rhythmic (phasic) or non-rhythmic (tonic) and is not a movement disorder or a sleep disorder in otherwise healthy individuals” [30].

#### 3.5.1. Case 1

The patient was a 49-year-old Caucasian male, a professional driver, who underwent a dental examination in which bruxism was detected [29,30,31]. He underwent several laboratory tests (in order to assess metabolic and hormonal functions), whole-night video-polysomnography (vPSG) adjusted to evaluating bruxism and parasomnias. PSG was evaluated in 30 s epochs, according to standard sleep criteria. Pathological events were evaluated according to the standards of the American Academy of Sleep [32]. The patient was also examined with a battery of scales and questionnaires: Athens Insomnia Scale (AIS), Epworth Sleepiness Scale (ESS), Berlin Questionnaire, STOP BANG questionnaire, Beck Depression Inventory (BDI), WHO Quality of Life–BREF (WHO QOL-BREF), Pittsburgh Sleep Quality Index (PSQI), Headache Impact Test–6 (HIT-6), Oral Behavior Checklist, Paris Arousal Disorder Severity Scale (PADSS) and Perceived Stress Scale-10 (PSS-10).

Medical history was positive for hypertension, psoriasis and Barrett’s esophagus. The patient reported a history of common nightmares and sleep terrors. He was also a long-term smoker. Medications included pantoprazole (40 mg), nebivolol (5 mg) and ramipril (5 mg). At presentation he complained of snoring, daytime fatigue, clenching and grinding of the teeth (during the day as well as night), pain located bilaterally in masseter muscles and area of temporomandibular joints, perceived increased masseter muscle tension and dissatisfaction with his quality of sleep. He identified his bruxism as one of the main causes of his subjectively reduced sleep efficiency.

Dental examination confirmed malocclusion (Angle’s class II), dental crowding, excessive overbite, teeth midline deviation and high Tooth Wear Index. During the examination, according to the Diagnostic Criteria for Temporomandibular Disorders (DC/TMD) guidelines, we found pain in both masseters and pain of both temporomandibular joints and also clicks in both temporomandibular joints during opening and closing of the mouth and during lateral and protrusive movements [31]. The Oral Behavior Checklist showed that the patient presented behaviors strictly connected with bruxism, such as bracing or thrusting of the mandible during the daytime. The patient knew about his bruxism and noticed an increased number and frequency of episodes after changing to a more stressful job. Dental examination together with Oral Behavior Checklist confirmed the high probability of severe sleep and awake bruxism.

Physical exam was within normal limits (WNL), apart from increased BMI (29.3). ESS score was 11 (mild daytime sleepiness). STOP BANG (patient scored 5 points) and Berlin questionnaires confirmed increased risk of Obstructive Sleep Apnea Syndrome (OSAS). PSS-10 indicated a higher level of perceived stress (21 points); BDI score of 9 was in the non-depressed range. Laboratory tests revealed only lowered concentration of HDL cholesterol, slight hypertriglyceridemia and slightly increased fasting glucose levels with normal levels of hormones and other metabolic parameters.

**Figure 1:**
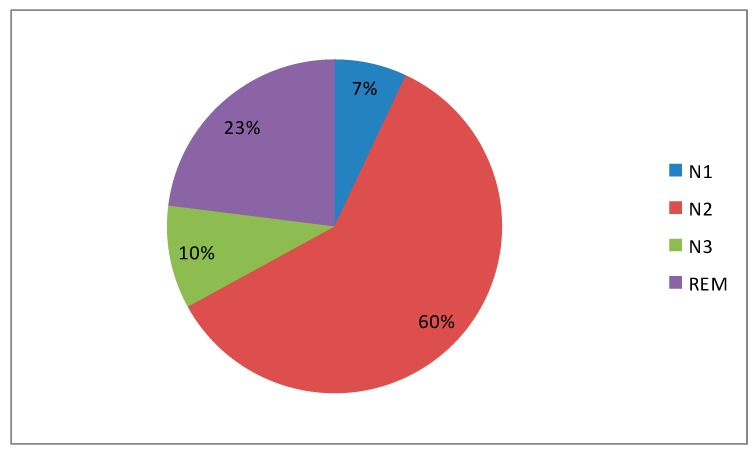
Sleep microstructure. N1, non-rapid eye movement stage 1; N2, non-rapid eye movement stage 2; N3, non-rapid eye movement stage 3; REM, rapid eye movement stage.

V-PSG confirmed the diagnosis of severe SB (Bruxism Episodes Index = 10.1/h, Bruxism Bursts Index = 12.8/h), with total count of 64 episodes lasting from 1.8 s up to 21.5 s (mean = 6.5 s). They often triggered EEG arousals. Suspected OSAS was not confirmed (AHI = 4.8/h, average SpO_2_ = 92.5%, Oxygen Desaturation Index = 5.5/h, average desaturation drop = 2.9%). Total Sleep Time (TST) was 379 min, Sleep Latency (SL) was 31 min and REM Latency (RL) was 160 min. Sleep stage distributions: N1 = 6.9%; N2 = 60.1%; N3 = 10%; REM sleep = 23%. Sleep moisturance is presented in Figure 1. Periodic limb movements (PLMs) were not evaluated due to technical limitations. PSG report is presented in Table 2.

The most unexpected and striking result was observed with the video recording. The patient had several episodes (total count = 8) of sexual activity, lasting from a few up to over a dozen seconds, which appeared during most sleep stages (N1, N2, and REM). The patient performed masturbation with his hand or with friction moves by pushing his loins against the quilt while lying in the prone position or on his side. The episodes were short, mostly lasting less than 20 s, and did not result in ejaculation. Notably, each of these sleep masturbation episodes was preceded by a bruxism episode with EEG arousal lasting for at least a few seconds. In most of the episodes, SB lasted throughout the whole sexsomnia episode. After awakening from all of these episodes, there was never any recall by the patient. There were no spontaneous arousals from N3 or N2 sleep, and REM-atonia was preserved. However, an episode of intermittent non-periodic myoclonic activity was recorded in N2, lasting 21 min and about 40 myoclonic movements in the left arm and hand were recorded. This episode ended with SB and an awakening. Mentioned episodes are presented in the Supplementary Figures S1–S3.

The patient was offered various treatments, including manual therapy jaw massages and an occlusal splint for the lower teeth arch with the goal of relaxing the masticatory muscles and decrease the number of SB/AW episodes, but he refused. When informed about the SB-triggered sexsomnia episodes, he confirmed that he was unaware and added that he would always ask for a single room in hotels.

#### 3.5.2. Case 2

The patient was a 38-year-old Caucasian male, who underwent a thorough dental examination in which bruxism was detected [29,30,31]. He underwent several laboratory tests (in order to assess metabolic and hormonal functions), whole-night vPSG adjusted to evaluating bruxism, OSAS and parasomnias. PSG was evaluated in 30 s epochs, according to standard sleep criteria. Pathological events were evaluated according to the standards of the American Academy of Sleep [32]. The patient was also examined with the same battery of scales and questionnaires that were mentioned in Case 1. Medical history was positive for masseter muscles hypertrophy and severe teeth damage. The patient reported a history of common nightmares and muscle cramps during sleep. He was also a long-term smoker. At presentation, he complained of loud snoring, daytime fatigue and massive teeth wear.

**Figure 2:**
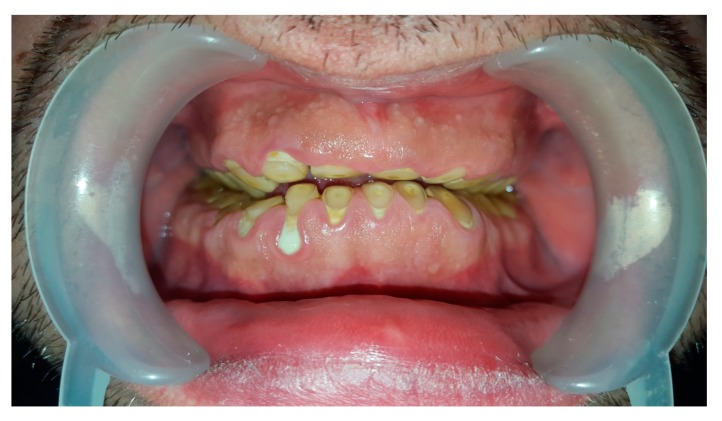
Tooth wear in presented bruxist patient.

Dental examination confirmed bilateral hypertrophy of masseter muscles and teeth wear of 4th stage in Tooth Wear Index (Figure 2).

Physical examination revealed enlarged palatine tonsils, apart from this examination was WNL, with Body-Mass Index 25.3. The ESS score was 16 and confirmed severe, excessive daytime sleepiness. STOP BANG confirmed high risk of OSAS, same with Berlin Questionnaire (2 categories positive). PSS-10 indicated a level of perceived stress within normal limits (19 points); BDI score of 7 was in the non-depressed range, while the PSQI score of 6 points confirmed a slightly decreased sleep quality. Laboratory tests revealed only lowered concentration of 25-hydroxycholecalciferol.

**Figure 3:**
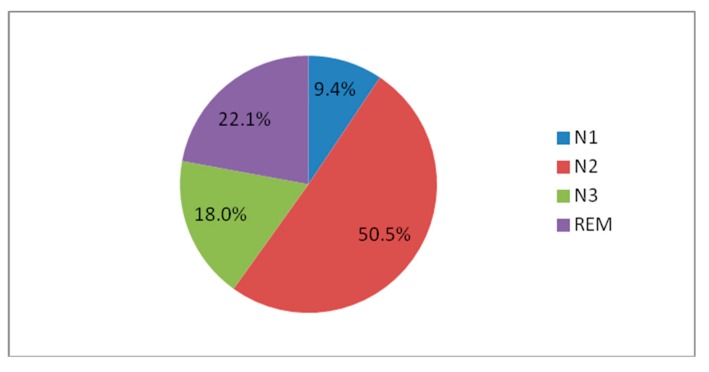
Sleep microstructure. N1, non-rapid eye movement stage 1; N2, non-rapid eye movement stage 2; N3, non-rapid eye movement stage 3; REM, rapid eye movement stage.

V-PSG confirmed the diagnosis of severe SB (Bruxism Episodes Index = 11.4 episodes/h, Bruxism Bursts Index = 3.1/h), with a total count of 69 episodes lasting from 2.4 s and up to 19.8 s (mean = 8 s). Most of the registered bruxism episodes were associated with respiratory events and spontaneous arousals. Also, the suspected OSAS was confirmed and evaluated as severe with the AHI = 33.5/h, average SpO_2_ = 92.2%, Oxygen Desaturation Index = 35.7/h, average desaturation drop = 6.1% and percentage of sleep time with blood oxygen saturation below 90% = 13.4%. Apnea to Bruxism Index = 7.6/h. Desaturations often triggered spontaneous arousals, with a total count of 57 arousals on the Arousal Index = 9.4. Total Sleep Time (TST) was 363.5 min, Sleep Latency (SL) was 10.5 min, and REM Latency (RL) was 59.5 min. Sleep stage distributions: N1 = 9.4%; N2 = 50.5%; N3 = 18%; and REM sleep = 22.1% (Figure 3). Pulse rate reached values between 60 and 103, with average = 75.6. Periodic limb movements (PLMs) were not evaluated due to technical limitations. PSG report is presented in Table 3.

In the video recording, we observed repeated episode of OSAS, SB and sexsomnia overlapping. There was a single period lasting about 17 min divided into several repeatable and similar episodes. The first episode started with hypopnea during the N2 stage in a supine position. When the patient reached a blood oxygen saturation level of 88%, spontaneous arousal occurred lasting 5 s and was accompanied by a mixed bruxism episode lasting 15 s. After 3 s of bruxism activity, the patient started to move his left arm and then masturbated with his dominant left hand. The whole motor activity of the arm lasted about 20 s and ended suddenly, right before the beginning of apnea episode. PSG recording with EEG of this event is presented in the Supplementary Figure S4. It started with blood oxygen saturation of 95% and lasted 35 s, during which the patient desaturated down to 86% and the apnea ended with the patient’s sudden gasp, accompanied by tonic bruxism episode (10 s) and again sexual motor activity lasting few seconds. There was a total period of 22 s of breathing and snoring, and then the next apnea appeared, lasting 33 s. Respiratory registration on the first two events are presented in the Supplementary Figure S5. We observed next 14 episodes which looked similar—first apnea/hypopnea appeared, during which saturation went down to about 89% and then breathing occurred with a desaturation drop, still decreasing down to 85–87%. During this desaturation drop bruxism and sexual activity appeared, but lasted only until the patient reached a saturation of 89–90% again. Then the activity stopped with the continuation of breathing for a few seconds until the next episode of apnea appeared. What is striking about this vPSG recording is the recurrence of these very similar episodes, each event happening within the same scheme, although during the last set of episodes normal breathing decreased between apneas and became shorter and shorter, from 28 s down to 9–10 s. After the last episode, the patient changed his position to non-supine and breathed normally, with no bruxism or sexsomnia until the next change of position. However, during the rest of the sleep time, no sexual activity was observed. Sexual activity was not leading to ejaculation, and it mostly appeared during the N2 stage or N1 stage, which was present after some spontaneous arousals evoked by respiratory events, similar to SB episodes. For the most of the time of sexual activity also the bruxism activity was present. The patient did not remember the episode. Respiratory registration of the whole described episode is presented in the Supplementary Figure S6.

The patient was offered treatment with continuous positive airway pressure (CPAP) according to the recommendations of the American Academy of Sleep Medicine. The first night with treatment resulted in a good response to treatment. Tonsillectomy was also advised. Dental treatment could be performed after OSA treatment and sleep bruxism management.

Both of the cases describe the coexistence of bruxism and sexsomnia. The mechanism of comorbidity of these both sleep disorders cannot be accurately determined because there are no other similar published clinical cases and what is more important, the mechanisms for generating sexsomnia instead of other parasomnia behaviors is still unknown. We suspect that the SB arousal can trigger sexual behaviors. However, these two cases (adult males, 39 and 49 years old) present the typical profile of sexsomnia that is more often connected with the male gender (approx. 75% in published cases), while there are notably no gender differences in SB, so that could have created a bias for sexsomnia episodes induced by SB arousals. Accordingly the presented cases, SB can be added to comorbid with sexsomnia sleep disorders, and the problem of comorbidity of these two issues should be studied more precisely.

## 4. Conclusions

Sexsomnia has still not been reported in the literature as often as other parasomnias. In most studies, it is reported as a case report or case series which is the main limitation. The coexistence of sexsomnia and other sleep-related disorders should be more thoroughly examined. This could help, both in sexsomnia, as well as in the treatment of other sleep-related disorders. Patients with established sexsomnia should undergo video-polysomnography to identify the possible coexistence of other sleep disorders, and its cause and effect reference to sexsomnia episodes. It would also be advised to perform screening of sexsomnia in patients with other sleep-related disorders, to establish a larger group of cases with such coexistence for further analysis and the improvement of treatment. The implementation of questions about the presence of possible abnormal sexual behavior during sleep and asking the patient’s partner may also be helpful.

## Figures and Tables

**Table 1:** Data extraction of selected studies.

Study, Year	Sleep Disorders That Co-occurred with Sexsomnia
Meira et al., 2016 [14]	Obstructive sleep apnea (OSA)
Khawaja et al., 2017 [15]	Obstructive sleep apnea (OSA)
Schenck et al., 2008 [16]	Obstructive sleep apnea (OSA)
Della Marca et al., 2009 [17]	Obstructive sleep apnea (OSA)
Ariño et al., 2014 [11]	Obstructive sleep apnea (OSA)
Gomis et al., 2016 [10]	Sleep-related eating disorder (SRED)
Yeh et al., 2016 [19]	Sleepwalking
Soca et al., 2016 [20]	Sleepwalking, sleep-related eating disorder, confusional arousals, sleep talking, REM sleep behavior disorder (RBD), obstructive sleep apnea (OSA)
Pelin et al., 2012 [21]	Sleepwalking and sleep talking
Cicolin et al., 2011 [22]	Sleepwalking, REM sleep behavior disorder (RBD), obstructive sleep apnea (OSA)
Shapiro et al., 2003 [23]	Sleepwalking, obstructive sleep apnea (OSA)
Irfan et al., 2018 [24]	Hypnic jerks, exploding head syndrome, obstructive sleep apnea (OSA)
Mioč et al., 2018 [25]	Hypnagogic hallucinations, sleep paralysis, night terrors, sleepwalking, sleep-related hyperomotor epilepsy

**Table 2:** Polysomnography report.

Index	Value
Sleep latency	30.7 min
REM latency	159.5 min
AHI	4.8/h
ODI	5.5/h
Average desaturation drop	2.9%
Average SpO_2_	92.5%
Minimum SpO_2_	88%
Bruxism Episodes Index	10.1/h
Bruxism Burst Index	12.8/h

REM, Rapid Eye Movement; AHI, Apnea/Hypopnea Index; ODI, Oxygen Desaturation Index; SpO_2_, Saturation.

**Table 3:** Polysomnography report.

Index	Value
Sleep latency	10.5 min
REM latency	59.5 min
AHI	33.5/h
ODI	35.7/h
Average desaturation drop	6.1%
Average SpO_2_	92.2%
Percentage of sleep time with saturation below 90%	13.4%
Bruxism Episodes Index	11.4/h
Bruxism Burst Index	3.1/h

REM, Rapid Eye Movement; AHI, Apnea/Hypopnea Index; ODI, Oxygen Desaturation Index; SpO_2_, Saturation.

## Supplementary Materials

The following are available online at http://www.mdpi.com/2077-0383/7/9/233/s1, Figure S1: Case 1. Sexsomnia episode on N1/N2 transition with bruxism activity preceding the episode, Figure S2: Case 1. Bruxism episode followed by sexsomnia in REM sleep, Figure S3: Case 1. Myoclonic activity—21 min episode, Figure S4: Case 2. EEG of a single sexsomnia event, Figure S5: Case 2. First two sexsomnia events, Figure S6: Case 2. Whole sexsomnia episode.
