# Supplementary material for: The Co-Occurrence of Sexsomnia, Sleep Bruxism and Other Sleep Disorders
Source: J Clin Med. 2018 Aug 23;7(9):233. doi: 10.3390/jcm7090233 (PMC6162860; doi:10.3390/jcm7090233)
Supplement: Supplementary file 1 [file jcm-07-00233-s001.pdf]

Journal of
Clinical Medicine

MDPI

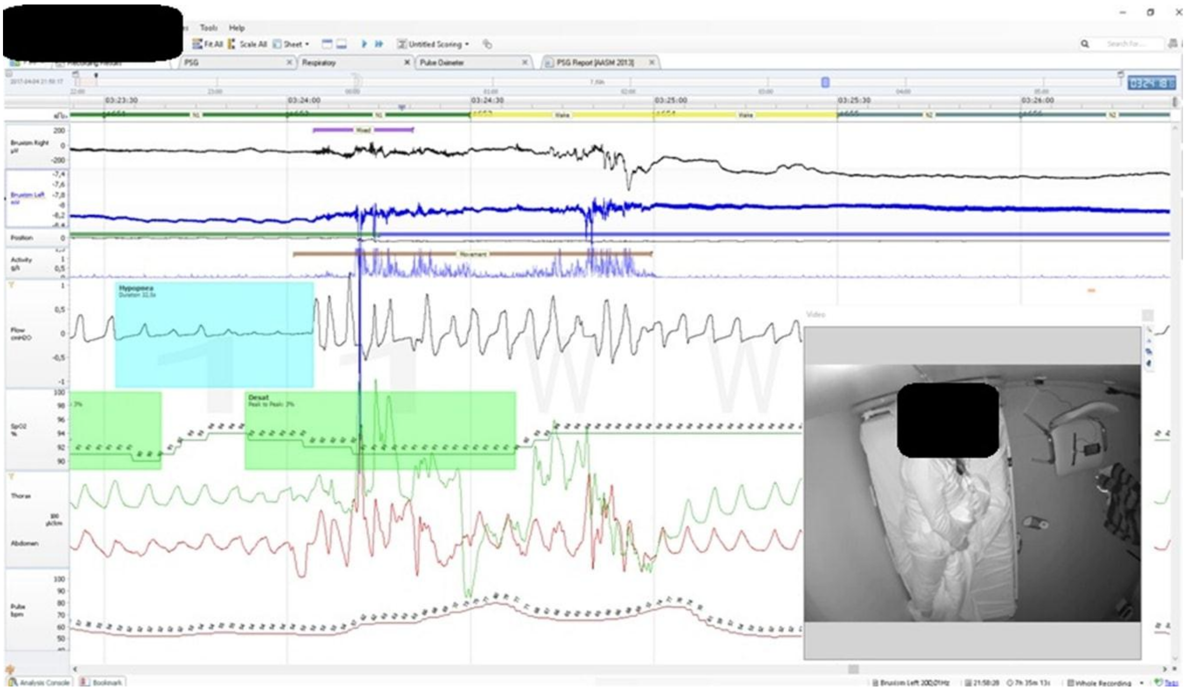

Figure S1. Sexsomnia episode on N1/N2 transition with bruxism activity preceding the episode.

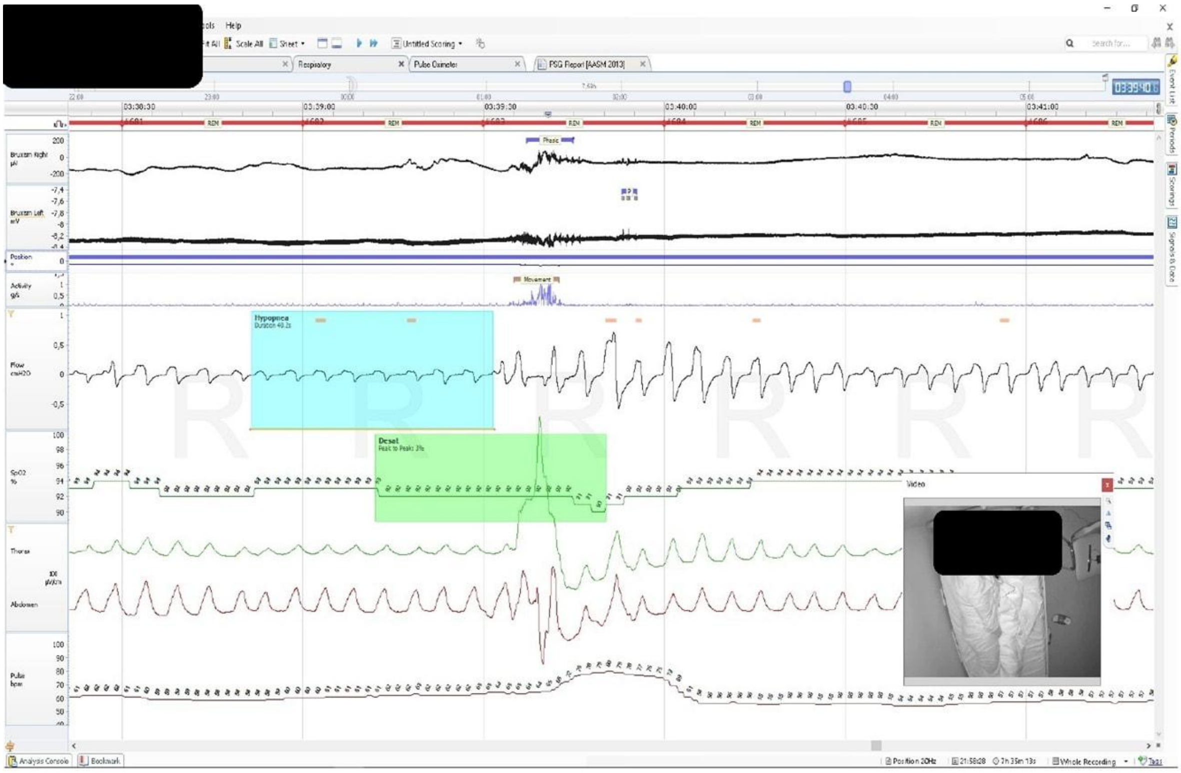

Figure S2. Bruxism episode followed by sexsomnia in REM sleep.

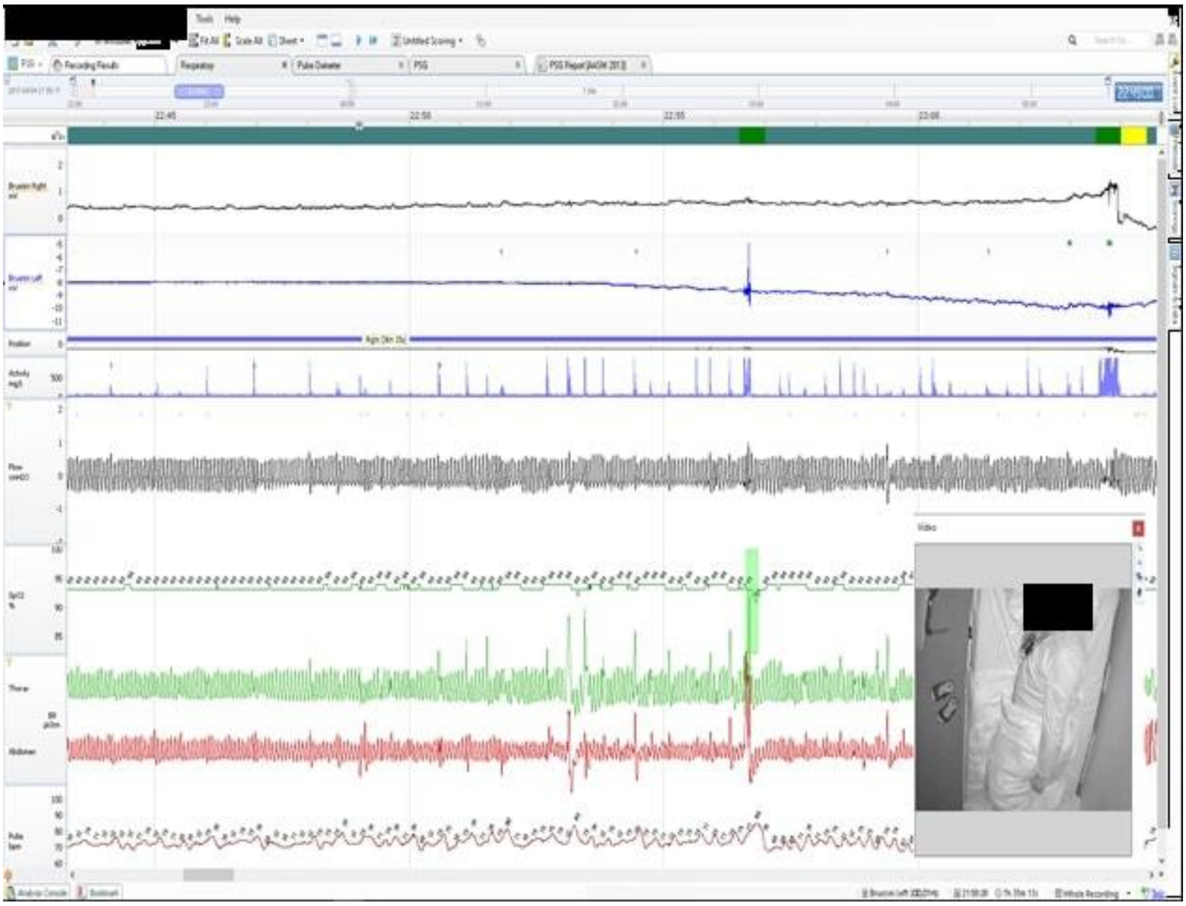

Figure S3. Myoclonic activity—21 min episode.

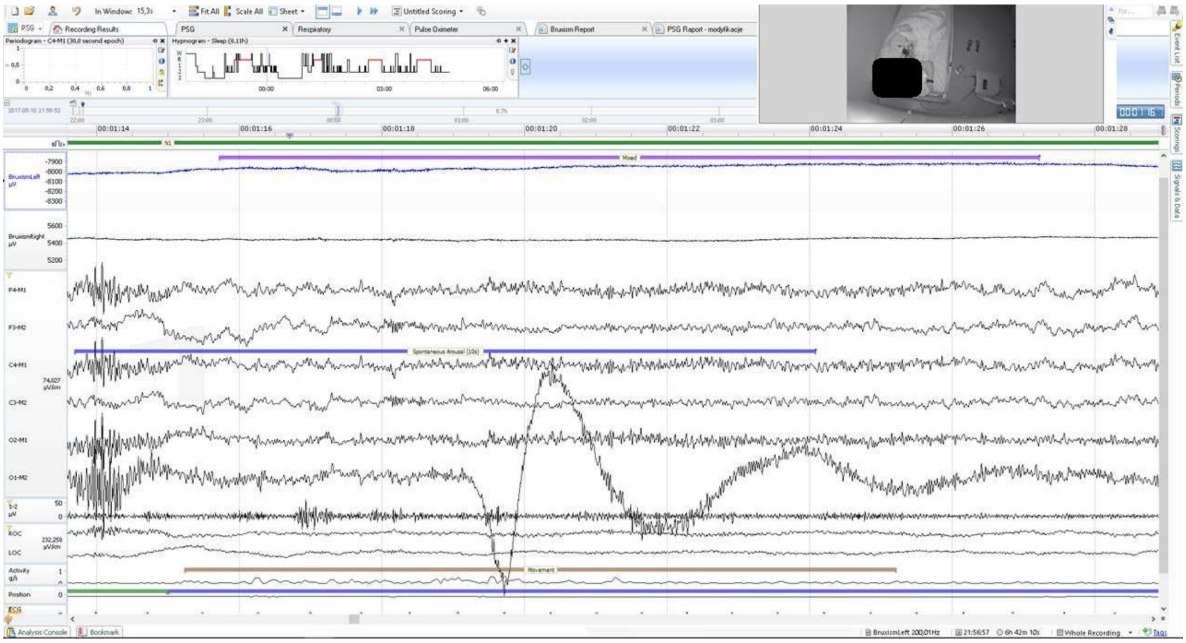

Figure S4. EEG of a single sexsomnia event.

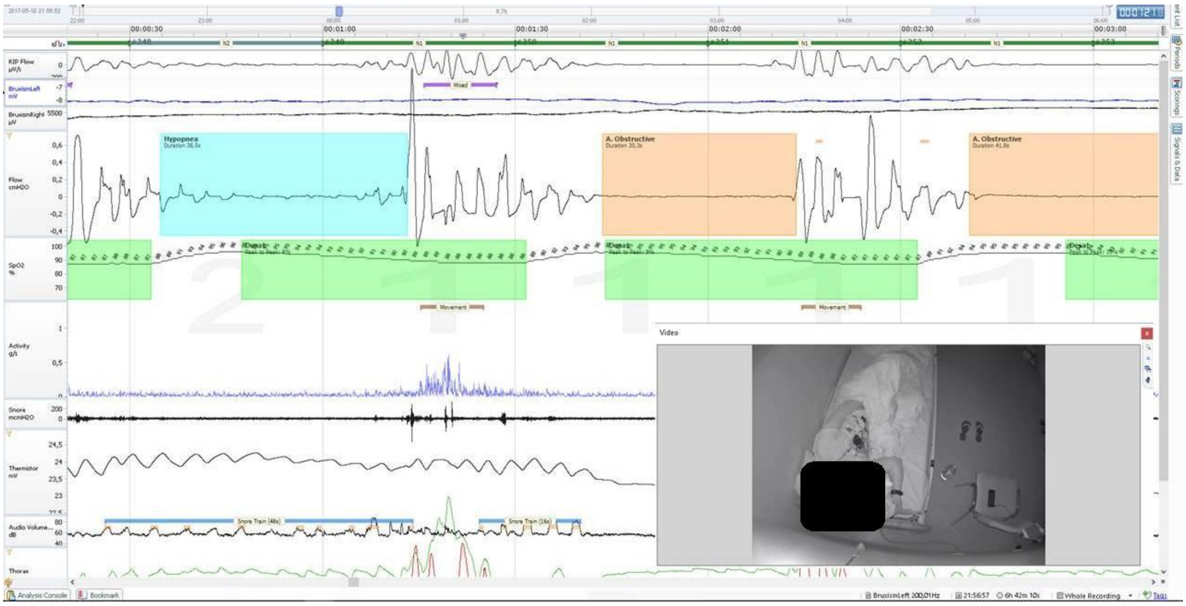

Figure S5. First two sexsomnia events.

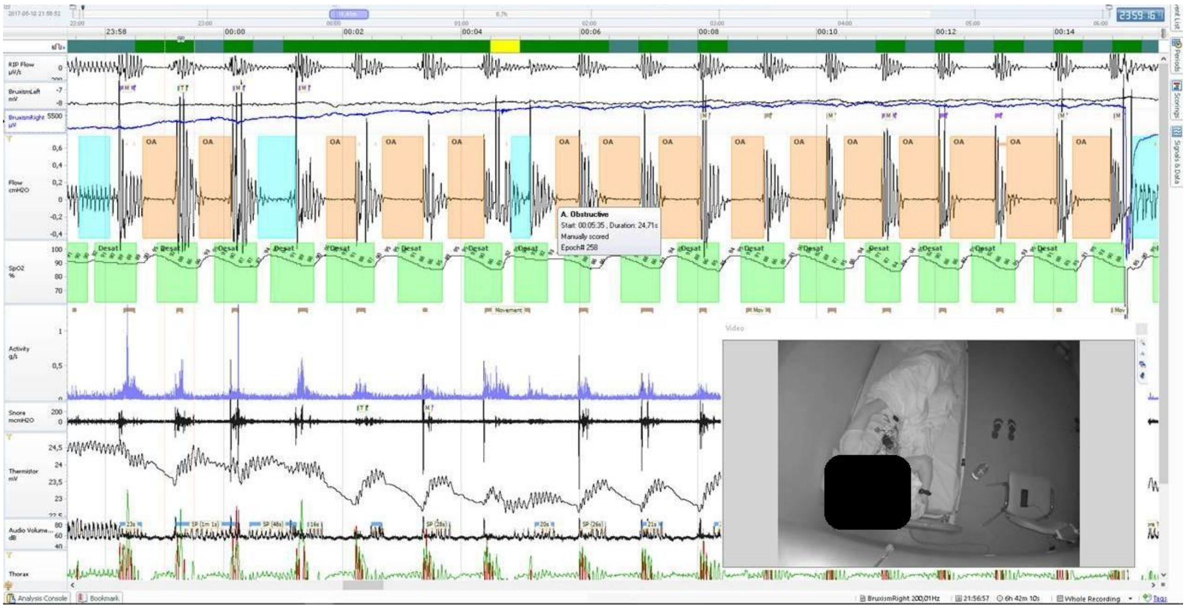

Figure S6. Whole sexsomnia episode.
